# Both p53 codon 72 Arg/Arg and pro/Arg genotypes in glioblastoma multiforme are associated with a better prognosis in bevacizumab treatment

**DOI:** 10.1186/s12885-020-07210-8

**Published:** 2020-07-29

**Authors:** Chiung-Chyi Shen, Wen-Yu Cheng, Chung-Hsin Lee, Xue-Jun Dai, Ming-Tsang Chiao, Yea-Jiuen Liang, Wan-Yu Hsieh, Tsuo-Fei Mao, Guo-Shi Lin, Shou-Ren Chen, Bai-Shuan Liu, Jun-Peng Chen

**Affiliations:** 1 Neurological Institute Head of Department of Neurosurgery Taichung Veterans General Hospital, Taichung, Taiwan; 2 Department of Physical Therapy, Hung Kuang University, No. 1650, Taiwan Boulevard Sec. 4 Taichung 407, Taichung, 43302 Taiwan; 3 Department of Medicine, National Defense Medical Center, Taipei, Taiwan; 4 Tri-Service General Hospital, National Defense Medical Center, Taipei, Taiwan; 5 Department of Game and Product Design, Chienkuo Technology University, Changhua city, Taiwan; 6 Basic Medical Education, Central Taiwan University of Science and Technology, Taichung, Taiwan; 7 Institute of Biomedical Sciences, National Chung Hsing University, Taichung, Taiwan; 8 Department of Neurosurgery, Neurological Institute, Taichung Tzu Chi Hospital, Taichung city, Taiwan; 9 Department of Neurosurgery, Zhangzhou Affiliated Hospital of Fujian Medical University, Zhangzhou city, China; 10 Department of Medical Imaging and Radiological Sciences, Central Taiwan University of Science and Technology, Taichung, Taiwan; 11 Biostatistics Task Force, Taichung Veterans General Hospital, Taichung, Taiwan

**Keywords:** Glioblastoma multiforme, Polymorphism, p53, Codon 72, Bevacizumab, SNP

## Abstract

**Background:**

It has previously been shown that bevacizumab, when added to chemotherapy, improved overall survival in several cancers. In glioblastoma multiforme (GBM), bevacizumab increased progression-free survival and it is widely used for tumor recurrence, though it has failed to improve overall survival (OS) in controlled trials. However, an effective biomarker for predicting the prognosis of bevacizumab treatment has yet to be identified. This study, therefore, aimed to retrospectively analyze the polymorphisms of p53 codon 72 and the clinical characteristics of GBM specimens from Taiwanese patients.

**Methods:**

The polymorphisms of p53 codon 72 in 99 patients with GBM treated at Taichung Veterans General Hospital in Taiwan from 2007 to 2017 were analyzed using direct DNA sequencing and PCR-RFLP analysis.

**Results:**

We found that among these GBM patients, the distribution of codon 72 polymorphisms was 28.3% for proline homozygotes (Pro/Pro), 38.4% for arginine homozygotes (Arg/Arg), and 33.3% for proline/arginine heterozygotes (Pro/Arg). Although the polymorphisms of p53 codon 72 were not directly associated with the overall survival of GBM, both the Arg/Arg and Arg/Pro genotypes were associated with significant benefits in terms of overall survival in patients treated with CCRT plus bevacizumab compared to patients treated with CCRT alone.

**Conclusions:**

This pilot study suggests that both the Arg/Arg and Arg/Pro genotypes of p53 codon 72 polymorphism may have value as independent prognostic or predictive parameters for bevacizumab treatment response and failure. Relatedly, the results of the study further demonstrate the utility of stratifying GBM patients according to bevacizumab sensitivity.

## Background

The tumor suppressor gene p53 is a core gene in the p53 signaling pathways, including those that guard the genome and prevent tumorigenesis by promoting cell growth arrest, DNA repair, senescence, and cell death, in addition to modulating autophagy and cancer metabolism [1, 2]. Hence, p53 is one of the most frequently mutated genes in human malignancies, with p53 mutations having been found in more than 50% of human cancers [3]. In addition to mutations that inactivate the functions of p53, many single nucleotide polymorphisms (SNPs) have been identified in the p53 gene that also influence the molecular function of the p53 protein as the guardian of the genome [4].

The p53 mutation database of the International Agency for Research on Cancer (IARC) lists 29 common polymorphisms in the non-coding region of TP53 [5]. Among these SNPs of the p53 gene, one well-known SNP that occurs on codon 72, the Arg72Pro polymorphism (rs1042522), is common. The p53 codon Arg72Pro is a C/G variation upstream of the p53 gene on human chromosome 17p13. According to past studies, the Pro72 variant (the Pro/Pro variant of p53 codon 72), meanwhile, is crucial for specifically activating the p53-dependent DNA repair genes in different cells, resulting in higher DNA-repair efficiency in vitro, suggesting that genomic instability is reduced in these cells [6]. However, the Arg72 variant (the Arg/Arg variant of p53 codon 72) protein encourages faster apoptosis and represses alteration more competently than the Pro72 protein [7]. Relatedly, while many case-control studies have investigated the association between p53 codon 72 Arg/Pro polymorphisms and glioma risk, those studies have provided inconsistent findings.

Bevacizumab is a humanized monoclonal immunoglobulin G1 antibody that is also the first antiangiogenic cancer therapy agent that has been used as a monotherapy or in combination with other anti-cancer agents for various types of cancer [8]. The possible mechanism by which bevacizumab achieves its effects is by targeting VEGF-A to prevent its interaction with VEGFR-1 and VEGFR-2 [9]. At present, bevacizumab is approved by the FDAs of more than 60 countries for the treatment of recurrent or progressive glioblastoma multiforme (GBM, WHO grade IV), although an effective biomarker for predicting the prognosis of bevacizumab treatment has yet to be found. In Taiwan, bevacizumab alone is used to treat adult patients with relapsed GBM who have undergone standard radiation therapy and have failed chemotherapy with temozolomide.

We therefore sought, in the present study, to determine the significance and context for appropriate clinical applications of p53 codon 72 variant polymorphisms based on specimens from 99 cases of GBM, in particular to determine whether there are any associations between p53 codon 72 polymorphisms and the effects of bevacizumab treatment for GBM. Our final goal was to find a novel angiogenesis-related predictive biomarker, of response to bevacizumab in recurrent GBM.

## Methods

### P53 polymorphism analysis by PCR amplification and direct sequencing

The extraction of genomic DNA was performed from frozen tumor tissues. For the p53 polymorphism analysis, target sequences (i.e., genomic DNA containing intron fragments) were amplified by polymerase chain reaction (PCR) using the following primer pairs: P1-F (5′- CCCACTTTTCCTCTTGCAGC-3′), P1-R (5′- CACTGACAGGAAGCCAAAGG-3′), P2-F (5′- AAgCTCCTgAggTgTAgACg-3′), P2-R (5′- gTTATAgggAggTCAAATAAgC-3′), P3-F (5′- TgCAgTgAgCTgAgATCACg-3′), P3-R (5′- AAACAgTCAAgAAgAAAACggC-3′), P4-F (5′- CA CAAgAATCACTTgAACCCC-3′), P4-R (5′- CAggCCAACTTgTTCAgTgg-3′). The DNA sequences of the PCR products were then determined using a DNA autosequencer (GeneAmp PCR System 2700 Thermal cycler; Applied Biosystems) (*N* = 76 cases of GBM) and a standard procedure [10]. All the heterozygous p53 polymorphisms were confirmed by direct sequencing of both strands.

We also analyzed an additional 23 GBM patients for p53 codon 72 using the polymerase reaction-restriction fragment length polymorphism (PCR-RFLP) method [11] due to its low cost and speed. Brief description of the method: a 366-bp fragment of the p53 gene was amplified from GBM genomic DNA using the forward primer (p53R72P-F) 5′-GTCCTCTGACTGCTCTTTTCACCCATCTAC-3′ and reverse primer (p53R72P-R) 5′-GGGATACGGCCAGGCATTGAAGTCTC-3′, (Supplementary Data Fig. S2) The PCR reaction mixture was pretreated at 95 °C for 10 min followed by 35 cycles at 95 °C for 1 min, 55 °C for 1 min, and 72 °C for 1 min. The final extension was conducted at 72 °C for 7 min. Enzyme digestion was conducted using 20 units of BstUI (New England Biolabs, Beverly, MA) and 10 μl of PCR product, and with a final volume of 20 μl. The reaction was incubated at 60 °C for 2 h, electrophoresed with 2% agarose gel and stained with ethidium bromide. The Arg/Arg homozygote of the p53 codon 72 variant was cleaved using BstUI and yielded 215- and 151-bp bands (Fig. 2e). The Pro/Pro homozygote was not cleaved using BstUI and yielded a single 366-bp band. The Arg/Pro heterozygote contained all three bands (366, 215, and 151 bp) following restriction digestion. In these specimens, we have chosen randomly 7 samples and confirmed the types of p53 codon 72 variant using DNA sequencing. The final all results of the p53 codon 72 variant were consistent (data not shown).

### Patients and follow-up

A total of 99 patients diagnosed with glioblastomas who underwent macroscopic total or near-total tumor resection at the Department of Minimally Invasive Skull Base Neurosurgery, Neurological Institute, Taichung Veterans General Hospital from 2007 to 2017 comprised the primary study cohort. All GBM patients were treated with surgical resection, received concurrent chemoradiotherapy with temozolomide (TMZ: 75 mg/m^2^/d) (CCRT), and adjuvant TMZ (150–200 mg/m^2^/d). Bevacizumab (10 mg/kg intravenously every 2 weeks until progression) was only used in patients with recurrent GBM. This retrospective review was exempt from the requirement for informed consent. The validation cohort consisted of 99 cases selected from the primary cohort based on the following criteria: (1) available follow-up data and samples and (2) a post-operative survival time of more than 1 month. This study was reviewed and approved by the committee on ethics of the Institutional Review Board of Taichung Veterans General Hospital. The obtained samples were frozen immediately after surgery with prior consent from the patients. The overall survival (OS) time was identified as the time from the operation to the date of death or censored at the date of the last follow-up examination. All the patients in the validation cohort were evaluated using the Karnofsky Performance Scale (KPS). The study end date was 31 March 2020.

### Statistical analysis

The demographic data was presented as the number (percentage) of patients for categorical variables and compared using the chi-squared test or Fisher’s exact test. OS outcomes were estimated using of the Kaplan-Meier method, and the differences in survival were determined using the log-rank test. All data were analyzed using the Statistical Package for Social Sciences (IBM SPSS version 22.0).

## Results

### Sequence comparison of p53 polymorphism variants in GBM specimens by direct DNA sequencing

**Fig. 1:**
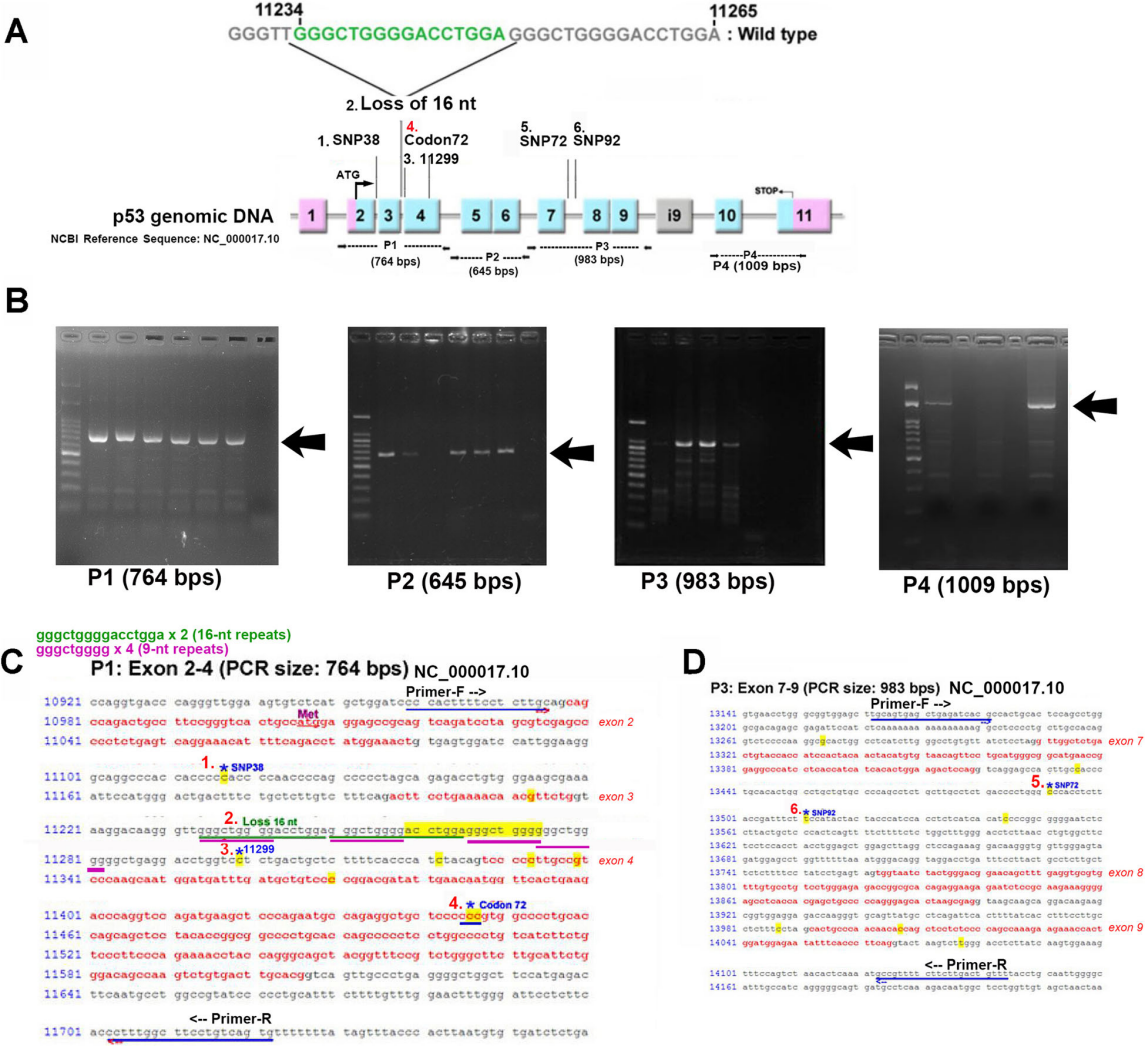
The location sites of 5 SNPs and a loss of nucleotide in p53 genomic DNA. **a** Schematic diagram of the p53 genomic DNA, the NCBI association number of which is NC_000017.10. The numbered boxes indicate different exons. We designed 4 pairs of primers (P1 to P4) to analyze the DNA sequence. We found 5 SNPs and a loss of 16-bp nucleotides, and assigned these variants the numbers 1–6. **b** Electropherogram of the 4 sections (P1-P4) of the p53 gene amplified via polymerase chain reaction (PCR) from a GBM tumor specimen. The PCR product sizes are indicated below each of the images. **c** The detailed sequences and locations of 4 SNPs in P1-PCR production. The red colored sequences of letters indicate exons, and the gray colored sequences of letters indicate introns. The numbers 1 to 4 indicate, respectively, SNP38 (No. 1), the 16-bp duplication polymorphism (rs17878362) (No. 2), SNP11299 (No. 3), and codon 72 (No. 4). The sequences of letters underlined in blue indicate primers. The sequence of letters underlined in green indicates the 16-bp duplication polymorphism. The sequences of letters underlined in purple indicate 4 9-bp repeats. **d** The detailed sequences and location of 2 SNPs in P3-PCR production. The red colored sequences of letters indicate exons, and the gray colored sequences of letters indicate introns. The numbers 5 and 6 indicate SNP72 (No. 5) and SNP92 (No. 6), respectively

To understand the genomic DNA mutant status of the p53 gene in the GBM specimens, p53 gene detection was performed by PCR followed by direct DNA sequencing. Figure 1a and b show the schematic diagram of the genomic p53 gene and the localization of PCR products in the p53 gene, respectively. According to the genomic DNA reference sequence of p53 (association number: NC_000017.10) from the National Center for Biotechnology Information (NCBI) database, we designed four pairs of primers, namely, the P1 (expected PCR DNA fragment size: 764 bps), P2 (expected PCR DNA fragment size: 645 bps), P3 (expected PCR DNA fragment size: 983 bps) and P4 (expected PCR DNA fragment size: 1009 bps) pairs, in order to analyze the over 3000 nucleotides of the p53 gene (Fig. 1a). Electropherograms of each PCR product size are shown in Fig. 1b. Among these PCR products, P2 and P3 covered the most common hotspots of p53 mutation (exons 5–8) [12, 13]. In conducting the DNA sequencing of dozens of GBM specimens and cell lines, we found 6 abnormal single nucleotide polymorphisms (SNPs) or losses of nucleotides, and we then gave each of them a sequence coding number (1–6). These numbers were 1. SNP38, 2. Loss of 16-nucleotides, 3. 11,299, 4. Codon 72, 5. SNP72, and 6. SNP92, respectively. Among them, Nos. 1–4 are located at the P1 PCR product (between exons 2–4 of the p53 gene), and Nos. 5–6 are located at the P3 PCR product (between the 7th intron of the p53 gene) (Fig. 1a). The detailed sequences and localizations of these abnormal points are shown in Fig. 1a, c, and d.

**Fig. 2:**
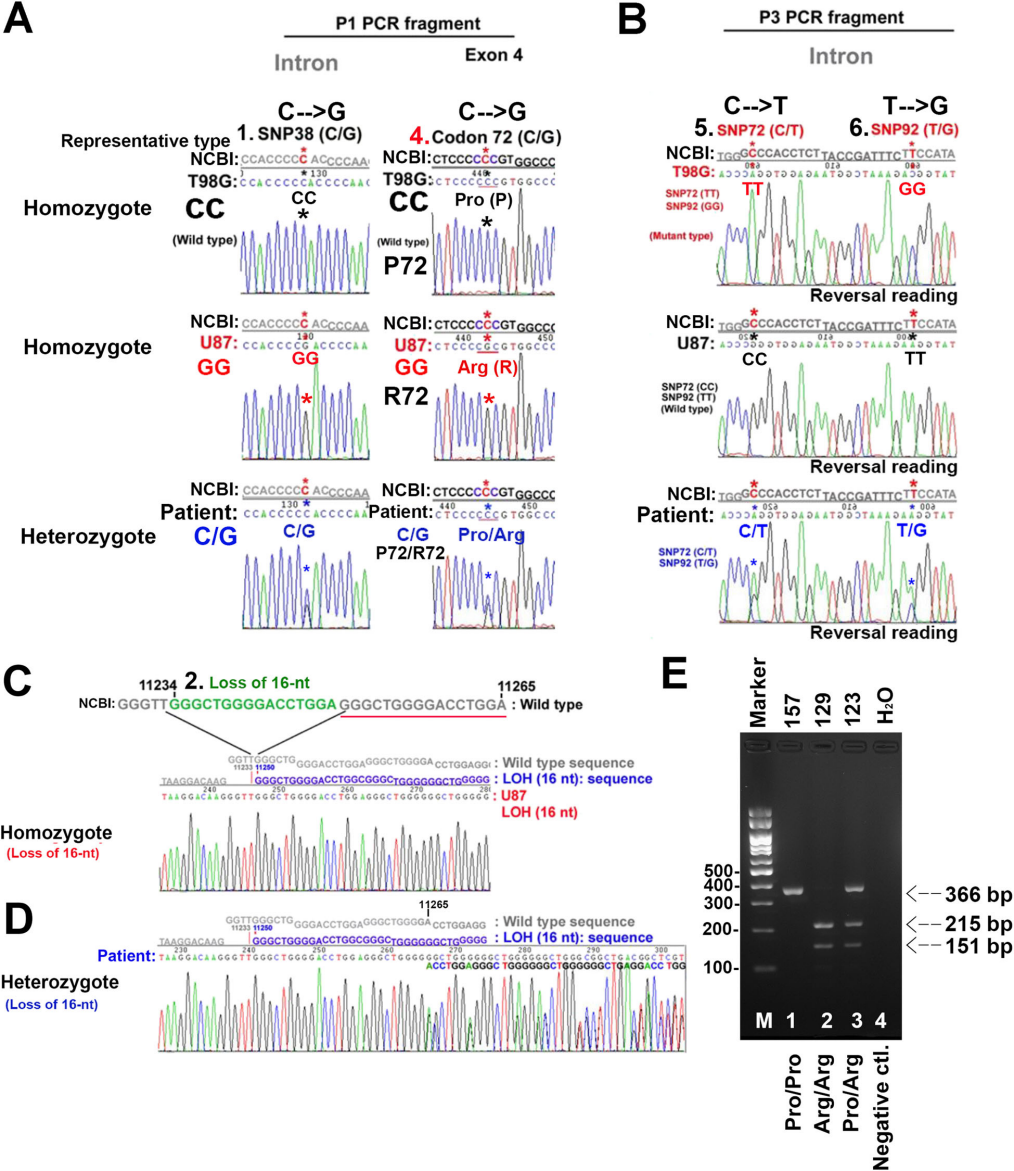
PCR products of P1 and P3 containing SNPs in the sequence analysis. **a** The P1 products of two SNPs, SNP38 (in an intron) and codon 72 (in an exon), of p53 were analyzed by DNA sequencing. Compared with the NCBI PubMed sequence, the sequencing data of the T98G cells presented as CC (single peak, asterisk) at both SNP 38 and codon 72, that is, as homozygous (upper layer). The sequencing data of the U87 cells presented as GG (single peak, asterisk) at both SNP 38 and codon 72, that is, as homozygous (middle layer). The sequencing data of the GBM specimen presented as CG (double peaks, asterisk) at both SNP 38 and codon 72, as heterozygous (lower layer). **b** Using the same sample and analysis as above, the P3 products of two SNPs, SNP 72 (in an intron) and SNP 92 (in an intron), of p53 were analyzed by DNA sequencing. **c** The 16-bp duplication polymorphism (rs17878362) of the U87 cells presents at a homozygote (loss of 16-nt). **d** A few of the GBM specimens presented as heterozygotes of the 16-bp duplication polymorphism resulting in the following sequence presenting a double peak. **e** BstUI PCR-RFLP analysis of the p53 codon 72 polymorphism. M, DNA ladder. Lane 1, Pro/Pro homozygote is not cleaved by BstUI and yield a single 366-bp band. Lane 2, Arg/Arg homozygote is cleaved by BstUI and yields 215- and 151-bp bands. Lane 3. Arg/Pro heterozygote contains all three bands (366, 215, and 151 bp) following restriction digestion

The types of these abnormal points are shown in Fig. 2. Both the No. 1 SNP38 and No. 4 Codon 72 are located at the P1 PCR product. More specifically, the No. 1 SNP38 is located in the 38th nucleotide of the 2nd intron between exons 2 to 3 (association number: NC_000017.10, no. 11117, single nucleotide polymorphism) (Fig. 1a, c). According to the NCBI provided sequence, this base position is a C nucleotide (CC, homozygote, single peak, Fig. 2a, asterisk). However, in our specimens from various individuals, this position was occupied by a G (GG, homozygote, single peak) or CG (heterozygote, two peaks, Fig. 2a asterisk). A similar situation of SNPs also exists in the No. 4 codon 72, No. 5 SNP72, and No. 6 SNP92, the differences being due to base substitutions. The focus is the No. 4 codon 72 (rs1042522) of the p53 gene where a single nucleotide polymorphism leads to the expression of a homozygote with arginine (CGC, Arg 72; R72), proline (CCC, Pro 72; P72), or P72/R72 heterozygote residues. However, we made a choice to halt the analysis of the DNA fragments of P2 and P4 because these DNA fragments were found to have no significant anomalies (*N* = 20, data not shown).

Interestingly, we found that in these four SNPs, a specific nucleotide resulted in certain linkages. For example, if the No. 1 SNP38 of T98G cells was identified as a CC genotype by direct DNA sequencing, the other three SNPs would be identified as follows: the No. 4 codon 72 must be a CC (Pro/Pro; P72; homozygote) genotype, the No. 5 SNP72 must be a TT genotype, and the No. 6 SNP92 must be a GG genotype. If the No. 4 codon 72 of U87 cells was identified as a GG (Arg/Arg; R72; homozygote) genotype by direct DNA sequencing, the other three SNPs would be identified as follows: the No. 1 SNP38 must be a GG genotype, the No. 5 SNP72 must be a CC genotype, and the No. 6 SNP92 must be a TT genotype (Fig. 2a, b). Furthermore, if any one of the above four SNPs was a heterozygote genotype, the other three SNPs also had to be a heterozygote genotype (Fig. 2a, b), such as a p53 haplotype. These specific coincidences always occur together, and were completely suitable for our DNA sequencing of the GBM specimens (*N* = 34, data not shown).

In addition, we also analyzed the p53 PIN3 A2 allele (16-bp duplication) at the 3rd intron of the p53 gene, which was the same as the 16-bp (base pairs) duplication polymorphism (rs17878362) [14]. We subsequently gave it a sequence coding No. 2 LO-16 nt. In our GBM specimens, the No. 2 LO-16 nt usual presented with homozygote genotyping (loss of double-strand DNA), which did not result in double peaks in the DNA sequencing of nucleotide No. 11250 (NCBI association number: NC_000017.10; Fig. 2c). However, we only detected a few heterozygote genotypes of No. 2 LO-16 nt, with these genotypes resulting in double peaks in the DNA sequencing of nucleotide No. 11265 (NCBI association number: NC_000017.10; Fig. 2d). Most importantly, we found that almost all of the GBM specimens had this loss of 16-bp (Table 1). Interestingly, we also found two kinds of nucleotide repeat elements in the 3rd intron of the p53 gene. One of them just the sixteen base pairs duplication polymorphism (Fig. 2c red sequences of letters and green underlining; Fig. 1c green underlining, green sequences of letters). The other one consists of four of the same 9-bp repeat sequences (gggctgggg) localized in this region (Fig. 1c, purple underlining, purple sequences of letters). The DNA repeat positions are likely to be used by the cell as a genome folding map [15]. To explore the available predicted biomarkers of bevacizumab treatment, we also analyzed the GBM tumor tissue status of a series of associated molecular genes, including mutations of the IDH gene and p53 genes, the protein expressions of p53 and hTERT, and the methylated status of MGMT promoter (Supplementary Data Tables 1, 2). Although most of the results indicated no statistically significant association in terms of distribution, the distribution of p53 wild-type status was 75% in the CCRT plus bevacizumab group (Supplementary Data Table 2), suggesting that one possible reason that this group benefited from the addition of bevacizumab was associated with the wild-type of p53 gene.

**Table 1:** The correlations among p53 codon 72 variants and various patient characteristics

	Patients (*n =* 99)		codon 72 (No.4)						*p* value
			CC (*n* = 28)		GG (*n* = 38)		CG (*n* = 33)		
	n	%	n	%	n	%	n	%	
**Age (*n*** **= 99)**									0.035*
> 60	58	58.59%	21	75.00%	23	60.53%	14	(42.4%)	
≦60	41	41.41%	7	25.00%	15	39.47%	19	(57.6%)	
**Gender (*n*** **= 99)**									0.819
Male	43	43.43%	13	46.43%	15	39.47%	15	45.45%	
Female	56	56.57%	15	53.57%	23	60.53%	18	54.55%	
**Tumor number (*n*** **= 99)**									0.442
Solitary	78	78.79%	20	71.43%	30	78.95%	28	84.85%	
Multiple	21	21.21%	8	28.57%	8	21.05%	5	15.15%	
**Tumor size (*n*** **= 99)**									0.138
> 3 cm	12	12.12%	6	21.43%	2	5.26%	4	12.12%	
≦3 cm	87	87.88%	22	78.57%	36	94.74%	29	87.88%	
**Tumor occurrence (*n*** **= 99)**									0.531
Primary	78	78.79%	20	71.43%	31	81.58%	27	81.82%	
Recurrence	21	21.21%	8	28.57%	7	18.42%	6	18.18%	
**Bevacizumab (*n*** **= 99)**									0.179
No used	45	45.45%	13	46.43%	21	55.26%	11	33.33%	
Used	54	54.55%	15	53.57%	17	44.74%	22	66.67%	
**LO-16 nt (*n =*** **76, DNA sequencing)** **(No. 2; rs17878362)**									0.804
Heterozygote loss	6	(7.9%)	3	(13.6%)	0	(0.0%)	3	(12.5%)	
Homozygote loss	70	(92.1%)	19	(86.4%)	30	30(100%)	21	(87.5%)	
**11,299 (No.3) (*n*** **= 76, DNA sequencing)**									0.007**
CC	65	85.53%	15	68.18%	30	100.00%	20	83.33%	
CA	8	10.53%	4	18.18%	0	0.00%	4	16.67%	
AA	3	3.95%	3	13.64%	0	0.00%	0	0.00%	

Chi-squared test. *p < 0.05, **p < 0.001, Statistical significance

**Fig. 3:**
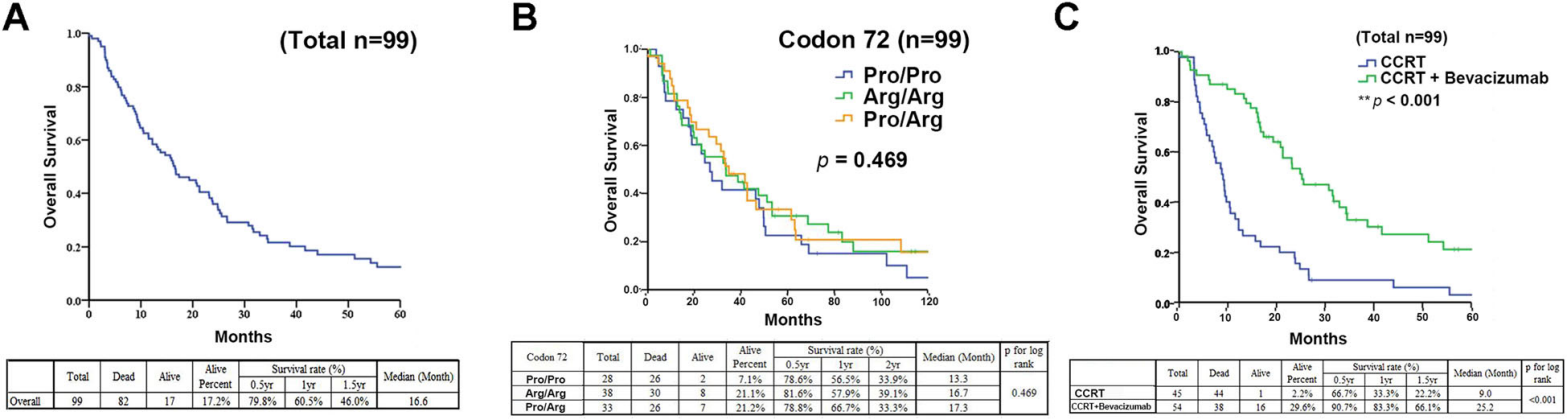
Kaplan-Meier curves for overall survival in patients treated with CCRT and CCRT plus bevacizumab. **a** Total overall survival duration. **b** Survival curves for GBM patients stratified by the p53 codon 72 genotypes. **c** Kaplan-Meier curves for the overall survival of patients during the CCRT standard treatment and CCRT plus bevacizumab treatment

To further investigate the relationship between the p53 polymorphisms and GBM, we undertook a retrospective cohort study of 99 cases of GBM. We employed the PCR of genomic DNA, using previously designed P1 primers (expected PCR DNA fragment size: 764 bps) and direct DNA sequencing to examine the p53 variants at codon 72. As shown in Table 1, the sex ratio of the analyzed GBM patients from whom the samples were taken was 56.6%: 43.4% (female: male), and the mean age of the patients was 55.6. We found that among these GBM patients, the distribution of codon 72 polymorphism was 28.3% for proline homozygotes (Pro/Pro, P72), 38.4% for arginine homozygotes (Arg/Arg, R72), and 33.3% for proline/arginine heterozygotes (Pro/Arg, P/R 72) (Table 1). The variants of the p53 polymorphisms did not correlate with patient age, patient gender, tumor number, tumor size, tumor occurrence, bevacizumab treatment, or LO-16-nt (*p* > 0.05) (Table 1). The 99 GBM patients investigated in the study died after a median follow-up of 16.6 months (range 11.31–21.87 months) (Fig. 3a). Next, we tried to evaluate the effects of the p53 codon 72 on the OS of these GBM patients. However, the OS analysis (*N* = 99) comparing the p53 codon variants did not show any significant results (Fig. 3b), with only a slightly better survival rate found to be associated with both the Arg/Arg and Pro/Arg variants. In addition, we also evaluated the effect of bevacizumab in combination with chemotherapy on the OS of GBM patients. The OS analysis comparing the patients treated with chemotherapy alone (*N* = 45) to those treated with a combination treatment including bevacizumab (*N* = 54) indicated that bevacizumab had a significantly positive effect (Fig. 3c). Indeed, in comparison with the concurrent chemoradiotherapy with temozolomide (CCRT) group, the group treated with CCRT plus bevacizumab group showed a significant (log-rank *p* < 0.001) improvement in median OS from 9 to 25.2 months (Fig. 3c). Our retrospective survey suggested that bevacizumab prolongs the OS of patients with recurrent GBM. However, these results still must be verified by a well-designed prospective randomized control trial.

### Genotype effect on overall survival after bevacizumab treatment

**Fig. 4:**
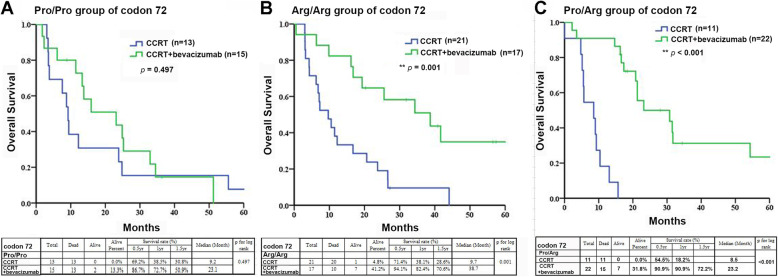
The genotypes of p53 codon 72 variants and Kaplan-Meier plots of OS for three groups of GBM patients are presented. **a** Comparison of the estimated OS for patients with tumor specimens with the p53 codon 72 Pro/Pro genotypes among those treated with CCRT and those treated with CCRT plus bevacizumab. **b** The estimated OS for patients with tumor specimens with the Arg/Arg genotypes of p53 codon 72 for CCRT and CCRT plus bevacizumab treatment. **c** The estimated OS for patients with tumor specimens with the heterozygous Arg/Pro genotypes of p53 codon 72 for CCRT and CCRT plus bevacizumab treatment. ** indicates *p* < 0.001

The overall survival data of all the GBM patients were included in the survival analysis. Based on the log-rank test and Kaplan-Meier survival curve analysis, overall survival was not significantly different between the CCRT group patients and the CCRT plus bevacizumab group patients with the codon 72 Pro/Pro genotype. Although the median survival of the patients treated with CCRT plus bevacizumab was 23.1 months compared to 9.2 months for the group treated with CCRT alone, the difference was not significant (*p* = 0.497, Fig. 4a). In contrast, in both the Arg/Arg and Pro/Arg genotype groups, the median survival durations of the patients treated with CCRT plus bevacizumab were greatly increased compared to that of the patients treated with CCRT only, with the differences being significant (*p* < 0.001, Fig. 4b, c). Overall survival was significantly increased in the CCRT plus bevacizumab treatment cohort among patients with both the Arg/Arg and Pro/Arg genotypes compared to those with the Pro/Pro genotype (Fig. 4). Similarly, in both the Arg/Arg (9.8 months) and Pro/Arg (9.6 months) genotype groups, the median survival duration of progression-free survival (PFS) of the patients treated with CCRT plus bevacizumab were greatly increased compared to the Pro/Pro genotype group (7.7 months) (Supplementary Data Fig. S1b).

**Fig. 5:**
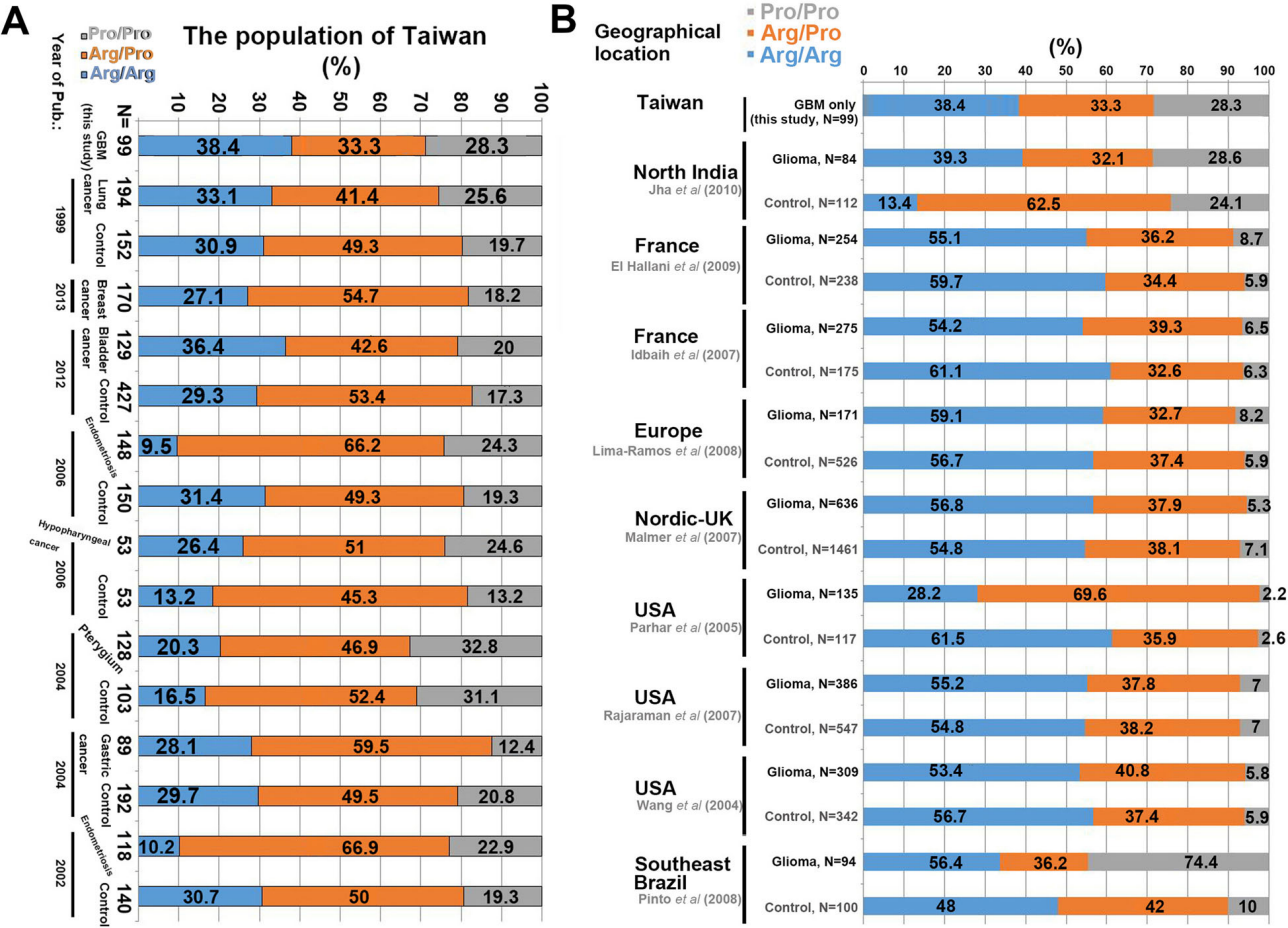
Comparison of the p53 codon 72 polymorphism distributions in normal control groups from various case/control studies in a limited region (Taiwan population only) (ref. 16–23) (**a**) GBM cases from around the globe (ref. 24–32) (**b**) and this study. The analyzed GBM cases showed a very similar distribution to the control groups in other studies of Caucasians (from the USA, Germany, and Europe). Studies from North India and Taiwan clearly showed a different allele distribution with a high frequency of the proline allele in comparison to Caucasians. N, number of analyzed cases

To clarify the differences in p53 codon 72 polymorphisms among people of different ethnicities in spite of the lack of a healthy control group, the distribution of the p53 codon 72 polymorphisms in the germline DNA of our patient cohort was compared to data for healthy controls in previously published studies, including healthy controls from the Taiwan population only [16–23], and for global glioma cases from the PubMed database [24–32] (Fig. 5). First, we only analyzed the relevant studies in Taiwan with p53 codon 72 data but did not distinguish the types of diseases. As shown in Fig. 5a, the distribution range for the Pro/Pro genotype of p53 codon 72 was found to be 12.4–32.8% (gray), while the ranges for the Arg/Arg and Arg/Pro genotypes were found to be 9.5–39.5% (blue) and 31.6–66.9% (orange color), respectively. Our data showed that the distribution of the p53 codon 72 genotypes in the GBM samples (Fig. 5a, lane 1) approached and met the expectations for the Taiwan population. Interestingly, among the global glioma cases (Fig. 5b), the distribution range of the Pro/Pro genotype of p53 codon 72 was only 2.2–8.7% in the Caucasian population, excluding India and Brazil, whereas the distribution range of the Pro/Pro genotype of p53 codon 72 in India was close to that in our GBM data, in spite of Indians being affiliated with the Caucasian population. Although the above data do not prove that p53 codon 72 is a risk factor for glioma, they show an obvious difference in the distribution of p53 codon 72 variant genotypes between Taiwan and Caucasian populations.

## Discussion

In this study, we only investigated the polymorphisms of p53 codon 72 and the tumor spectrums of Taiwanese patients (total number = 99) with GBM, ignoring the question of whether or not the p53 gene had a mutation in any given case. In investigating the distribution of p53 codon 72 genotypes in our Taiwanese population of GBM patients, we found that the genotypes included Pro/Pro (28.3%), Arg/Arg (38.4%), and Arg/Pro (33.3%) genotypes. The results showed that all the genotypes of p53 codon 72 are not directly associated with the overall survival of GBM patients. However, the addition of bevacizumab treatment significantly prolonged overall survival and progression-free survival compared with those of the patients treated with CCRT alone (Fig. 3c and Supplementary Data Fig. S1). Interestingly, in analyzing the different treatments, we found that the retention of the p53 codon 72 arginine allele in the tumor tissue of both the Arg/Arg homozygous and Pro/Arg heterozygous GBM patients was associated with significantly prolonged overall survival. Such results are very unique and rare. Consequently, the results presented here still require further confirmation in a larger sample of patients.

Glioblastoma multiforme is a multifactorial and complex disease and is closely associated with the interactions between environmental factors and genetic backgrounds. Past studies of the p53 gene have displayed a large amount of information regarding the complexity of its function in and regulation of the brain tumor process. Mutations in the p53 gene occur frequently in many kinds of cancerous tumors, including brain tumors. More specifically, approximately 10% (of hematopoietic malignancies) to 50–70% of all other human cancers are found to have somatic mutations in the p53 gene [33], with approximately 25% of gliomas carrying mutations in the p53 gene [34]. Not only are the p53 mutants able to attenuate cancer cell responses to radiotherapy; rather, they can also trigger chemoresistance [35, 36], even driving resistance to antiangiogenic therapies targeting the GBM vasculature [37]. Due to other well-known prognostic markers already being in use, we sought to determine the significance and context for appropriate clinical applications of p53 status as a marker in our cohort. Our retrospective analysis results showed that some GBM patients who benefited from bevacizumab treatment were likely to carry the wild-type of the p53 gene (Supplementary Data Tables 1, 2).

In past research about the p53 gene polymorphisms in the non-coding region, the 16-bp duplication polymorphism (rs17878362) within intron 3 has been widely analyzed as a possible cancer susceptibility modifier. The Ins allele variant of the p53 intron 3 16-bp duplication polymorphism is associated with a significantly increased risk of breast cancer [38]. One possible reason for this is that the 16-bp Ins allele (duplication, A2A2) leads to lower level of p53 transcript, suggesting that extra-long polymorphisms are likely to cause an alteration in the processing of messenger RNA (mRNA), to the extent of even being translated into a defective protein, leading to a decrease in the p53-mediated apoptosis of tumor cells [38, 39]. The haplotypes with mutant alleles of the p53 (A2) gene were previously found to be associated with an increased risk for tumorigenesis. Moreover, in a comparison of different population groups, increased cancer risk was associated with A2A2 carrier in Indian, Mediterranean, and Northern European populations but not in the Caucasian population of the United States [40]. Being an A2A2 carriers is also a high-risk factor for breast and colorectal cancers, but not for lung cancers [40]. In our results, we analyzed the distribution of the genotype (rs17878362) carriers of the duplicated allele and found that those in which a deletion was recorded (A1A1) (92.1%) constituted the most prevalent genotype in the GBM patients, being far more common than those in which an insertion was recorded (A2A1) (7.9%) among heterozygote carriers, while not a single homozygote carrier (A2A2) was found. Although these results were not statistically significant, they still represent the distribution of A2A2 genotypes (rs17878362) in the Taiwanese population and indicate the tumor-specific effects of GBM.

Many case-control studies have investigated the association between p53 codon 72 Pro/Arg polymorphism, but those studies have provided inconsistent findings. Nonetheless, more and more meta-analysis evidence obtained by pooling all the currently available case-control studies to estimate the effect and distribution of p53 codon 72 Arg/Pro polymorphism on cancer susceptibility has indicated that the polymorphism is not associated with overall cancer odds in several populations, including Iranian [41] and Chinese [42] colorectal cancer patients [43]. However, others studies have indicated the opposite, that is, that the polymorphism is associated with increased risk of several malignancies associated with viral infections, including in Caucasian (prostate cancer) [44], European (high-grade glioma; Pro 72) [45], Indian (cervical cancer) [46], Chinese (cervical cancer) [47], and Japanese (hepatocellular carcinoma) populations [48], implying that important ethnic and tumor type differences may exist regarding the cancer susceptibility of p53 codon 72 polymorphisms. Although our data could not show whether a given p53 codon 72 polymorphism is a risk factor, such polymorphisms may have an influence on GBM susceptibility in combination with certain other elements. Also, our data regarding the distribution of genotypes in codon 72 showed various shared and unshared GBM characteristics between Caucasian and Taiwanese patients (Fig. 5). This is the first report on the p53 codon 72 polymorphism spectrum and clinical characteristics of a large series of high-grade Taiwanese glioma patients.

Recent studies regarding the two genetic variants of p53 codon 72 (Pro 72 and Arg 72) have indicated that the two variants have different functional activities in the molecular mechanism. Pro 72 has significantly higher levels of BPDE (benzo [a] pyrene-7,8-9,10-diol epoxide)-induced apoptosis index compared to Arg 72 in primary lymphocytes [49]. The Arg 72 protein encourages (15-fold) faster apoptosis [50] and represses genetic alteration more competently than the Pro 72 protein [7]. Furthermore, cancer patients harboring wild type p53 and Arg 72 genotypes and patients with mutant p53 in combination with Pro 72 genotypes had better response rates, as well as better overall and progression-free survival [51]. Also, the p53 codon 72 variants show different affinities in terms of protein-protein interactions, resulting in a different half-life and phosphorylation of the p53 protein. The phosphorylation at Ser-20 of the p53 protein escapes from degradation by MDM2 (as E3 ubiquitin-protein ligase for p53), leading to stabilization of the p53 protein [52]. A recent study further revealed that both VEGF-A and MMP-9 are significantly elevated in mammary glands from R72 compared to P72 mice in a humanized p53 mouse model, with the R72 mice also showing a significantly higher amount of CD31+ blood vessels [53]. Similarly, increased angiogenesis has been observed in glioma model mice, in which the COX-2 and CCL2 pathways promote gliomagenesis by directly supporting the systemic development of myeloid-derived suppressor cells (MDSC) and their accumulation in the tumor microenvironment, where they limit cytotoxic T lymphocyte infiltration [54]. However, this vicious process can be blocked or slowed down in glioma-bearing COX2-deficient and CCL2-deficient mice, through treatment with COX-2 inhibitors, and through MDSC-mediated immunosuppression [54]. Inferring from this mechanism, it seems likely that the R72 variant that induces angiogenesis and vasodilation is susceptible to treatment with bevacizumab.

In February of 2004, the US FDA approved the use of bevacizumab as a first- or second-line treatment for patients with metastatic carcinoma of the colon or rectum, and since that time, finding effective and suitable cancer biomarkers has been one of the important steps in the treatment of cancers [55]. These biomarkers usually are not only expressed abnormally but are involved in the vital biological processes, including angiogenesis, differentiation, and growth [55]. Several predictive genomic biomarkers for bevacizumab have been identified in various indications, including pH-weighted amine chemical exchange saturation transfer echoplanar imaging [56], soluble carbonic anhydrase IX [57], and apelin/APLN [58], among others, while none has been prospectively validated yet [59]. The lack of any ability to identify patients who will benefit a priori, that is, the absence of a predictive biomarker, means that nearly every patient is treated with the antibody, in spite of not knowing which patients would be more likely to derive any benefit from it. It is interesting that several clinical data have confirmed that bevacizumab has clinically meaningful efficacy and an acceptable safety profile in Asian populations, as well as in global populations [60–62], suggesting our results consistent with previous reporting data.

The severe difficulty of determining the exact effects of different variants is likely to be influenced by their multiple roles within individual microenvironments as compared with tumor intrinsic changes such as oncogene mutations of amplification [59]. Hence, we conducted the current study of clinical experiences with the hope of elevating the benefits of bevacizumab treatment. Upon a patient beginning treatment with bevacizumab, the dose of bevacizumab given is determined according to the standard dose. During this period, we will continue to observe the status of the tumor through MRI. If the patient has no recurrence for more than half a year, we consider providing a half dose of the bevacizumab treatment (doubling the period over which it is given) until the patient experiences a recurrence. One theory regarding the effects of bevacizumab suggests that they may be related to the extreme hypoxic microenvironment activating the neo-angiogenic network [63]. In spite of this, we could not put forward a specific statistical significance for the above-mentioned mechanisms in the process of bevacizumab treatment. Further studies are required to elucidate the effects of these molecular mechanisms.

## Conclusions

We conclude that among the Taiwanese population of glioma patients, the p53 mutant status and p53 codon 72 polymorphisms had relatively little prognostic influence in GBM patients. The bevacizumab plus CCRT treatment significantly prolongs the overall survival of GBM patients compared with the CCRT treatment alone. Relatedly, we found a significant difference in the overall survival of GBM treated with CCRT plus bevacizumab compared with GBM treated with CCRT alone that was dependent on the different p53 codon 72 genotypes, with both the Arg/Arg and Pro/Arg genotypes being associated with a better prognosis than the Pro/Pro genotype. Despite this, however, there is still no reasonable explanation for the benefits of bevacizumab treatment being connected with p53 codon 72 polymorphism variants. Nonetheless, the variants of the p53 codon 72 polymorphism are likely to worth considering as a predictive biomarker before providing bevacizumab treatment. Further studies with larger population sizes are still warranted to confirm the results of the present study.

## Supplementary information

**Additional file 1.**

## Data Availability

All relevant data have been uploaded to DRYAD and can be accessed using the following link: https://datadryad.org/stash/share/vlsUBJojP5DP0aT8WleUEjZlV0NpAWzNPkY7DOOWCNg

## Abbreviations

GBM: Glioblastoma multiforme
SNP: Single nucleotide polymorphisms
mRNA: Messenger RNA
CCRT: The concurrent chemoradiotherapy with temozolomide treatment
BPDE: Benzo [a] pyrene-7,8-9,10-diol epoxide
MDSC: Myeloid-derived suppressor cells
PCR-RFLP: Polymerase chain reaction-restriction fragment length polymorphism
