# Supplementary material for: Both p53 codon 72 Arg/Arg and pro/Arg genotypes in glioblastoma multiforme are associated with a better prognosis in bevacizumab treatment
Source: BMC Cancer. 2020 Jul 29;20:709. doi: 10.1186/s12885-020-07210-8 (PMC7391574; doi:10.1186/s12885-020-07210-8)
Supplement: Supplementary file 1 — Additional file 1. [file 12885_2020_7210_MOESM1_ESM.docx]

| Table 1. Characteristics of IDH status, p53 gene status, hTERT, and MGMT methylation in GBM patients with three types of p53 codon 72 polymorphism. | | | | | | | | | | | | |  |
| --- | --- | --- | --- | --- | --- | --- | --- | --- | --- | --- | --- | --- | --- |
|  | Patients | |  | p53 codon 72 | | | | | | | | *p* value | |
|  |  |  |  | CC | |  | GG | |  | CG | |  |  |
|  | n | % |  | n | % |  | n | % |  | n | % |  |  |
| IDH gene status |  |  |  |  |  |  |  |  |  |  |  | 0.390 | |
| Wild type | 21 | (45.7%) |  | 8 | (61.5%) |  | 8 | (38.1%) |  | 5 | (41.7%) |  | |
| Mutant type | 25 | (54.3%) |  | 5 | (38.5%) |  | 13 | (61.9%) |  | 7 | (58.3%) |  | |
| p53 IHC |  |  |  |  |  |  |  |  |  |  |  | 0.549 | |
| Negative | 5 | (50.0%) |  | 2 | (50.0%) |  | 3 | (60.0%) |  | 0 | (0.0%) |  | |
| Positive | 5 | (50.0%) |  | 2 | (50.0%) |  | 2 | (40.0%) |  | 1 | (100.0%) |  | |
| p53 gene status |  |  |  |  |  |  |  |  |  |  |  | 0.184 | |
| Wild type | 14 | (41.2%) |  | 3 | (30.0%) |  | 4 | (30.8%) |  | 7 | (63.6%) |  | |
| Mutation | 20 | (58.8%) |  | 7 | (70.0%) |  | 9 | (69.2%) |  | 4 | (36.4%) |  | |
| hTERT IHC |  |  |  |  |  |  |  |  |  |  |  | 0.482 | |
| Negative | 7 | (25.0%) |  | 1 | (12.5%) |  | 4 | (36.4%) |  | 2 | (22.2%) |  | |
| Positive | 21 | (75.0%) |  | 7 | (87.5%) |  | 7 | (63.6%) |  | 7 | (77.8%) |  | |
| MGMT promoter |  |  |  |  |  |  |  |  |  |  |  | 0.683 | |
| Un-methylation | 21 | (47.7%) |  | 7 | (58.3%) |  | 9 | (42.9%) |  | 5 | (45.5%) |  | |
| Methylation | 23 | (52.3%) |  | 5 | (41.7%) |  | 12 | (57.1%) |  | 6 | (54.5%) |  | |
| IHC immunohistochemistry. Chi-square test. **p*<0.05, ***p*<0.01 | | | | | | | | | | | | |  |

| Table 2. Demographic data of patients included in each histological expression and gene status group of the indicated gene. | | | | | | | | | |
| --- | --- | --- | --- | --- | --- | --- | --- | --- | --- |
|  | GBM Patients | |  | CCRT only | |  | CCRT+Bevacizumab | | *p-*value |
|  | n | % |  | n | % |  | n | % |  |
| IDH gene status |  |  |  |  |  |  |  |  | 0.388 |
| Wild type | 21 | (45.7%) |  | 12 | (54.5%) |  | 9 | (37.5%) |  |
| Mutant type | 25 | (54.3%) |  | 10 | (45.5%) |  | 15 | (62.5%) |  |
| P53 IHC |  |  |  |  |  |  |  |  | 0.524 |
| Negative | 5 | (50.0%) |  | 4 | (66.7%) |  | 1 | (25.0%) |  |
| Positive | 5 | (50.0%) |  | 2 | (33.3%) |  | 3 | (75.0%) |  |
| P53gene status |  |  |  |  |  |  |  |  | 0.042* |
| Wild type | 14 | (41.2%) |  | 8 | (30.8%) |  | 6 | (75.0%) |  |
| Mutant type | 20 | (58.8%) |  | 18 | (69.2%) |  | 2 | (25.0%) |  |
| hTERT IHC |  |  |  |  |  |  |  |  | 0.418 |
| Negative | 7 | (25.0%) |  | 3 | (18.8%) |  | 4 | (33.3%) |  |
| Positive | 21 | (75.0%) |  | 13 | (81.3%) |  | 8 | (66.7%) |  |
| MGMT promoter |  |  |  |  |  |  |  |  | 1.000 |
| Un-Methylation | 21 | (47.7%) |  | 11 | (50.0%) |  | 10 | (45.5%) |  |
| Methylation | 23 | (52.3%) |  | 11 | (50.0%) |  | 12 | (54.5%) |  |
| Chi-square test. **p*<0.05, ***p*<0.01 | | | | | | | | | |

| Progression free survival  a. | | | | | | | | |
| --- | --- | --- | --- | --- | --- | --- | --- | --- |
|  | Total | PD | Non-PD | Alive  Percent | Survival rate (%) | | | Median (Month) |
|  |  |  |  |  | 0.5yr | 1yr | 1.5yr |  |
| Bevacizumab | 54 | 51 | 3 | 5.6% | 68.2% | 39.8% | 28.4% | 9.1 |


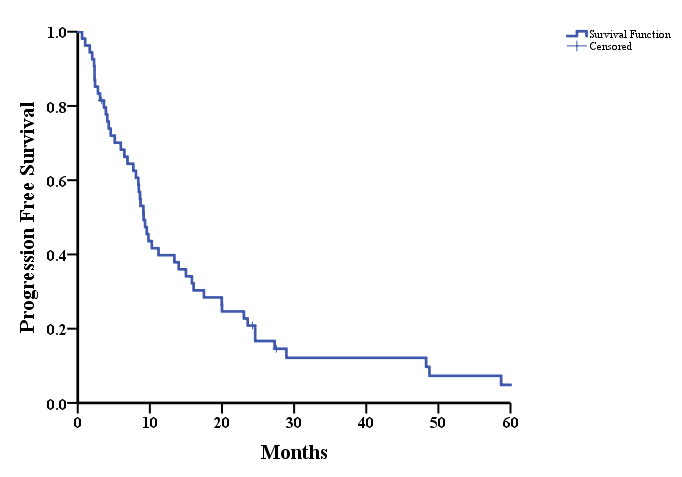


| Progression free survival  b. | | | | | | | | | | |
| --- | --- | --- | --- | --- | --- | --- | --- | --- | --- | --- |
| Codon 72 | Total | PD | Non-PD | Alive  Percent | Survival rate (%) | | | Median (Month) | p for log rank |  |
|  |  |  |  |  | 0.5yr | 1yr | 2yr |  |  |  |
| Pro/Pro | 15 | 14 | 1 | 6.7% | 60.0% | 33.3% | 6.7% | 7.7 | 0.224 |  |
| Arg/Arg | 17 | 17 | 0 | 0.0% | 64.7% | 41.2% | 29.4% | 9.8 |  |  |
| Pro/Arg | 22 | 20 | 2 | 9.1% | 76.8% | 43.2% | 24.0% | 9.6 |  |  |


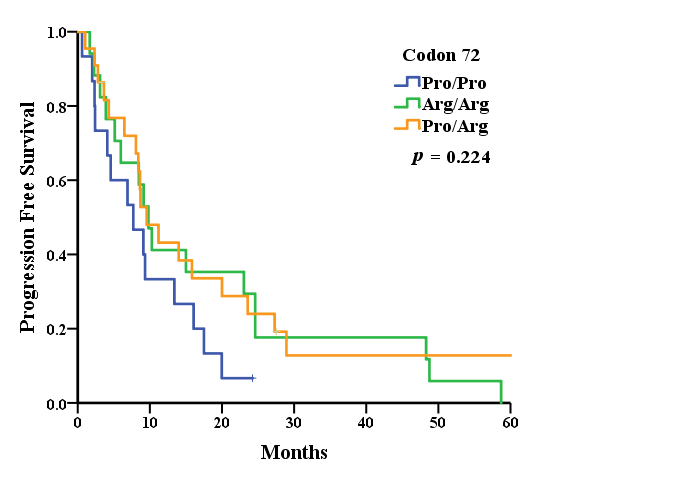


Supplementary data Fig S1. Survival analyses. Progression-free survival (PFS) and three genotypes of p53 codon 72 variants for all randomly assigned patients were analyzed with Kaplan-Meier methods. (a) Kaplan-Meier plots showing progression-free survival (PFS). (b) Kaplan-Meier plots showing progression-free survival (PFS), according to three genotypes of p53 codon 72 variants for all GBM patients. PD: progression disease.


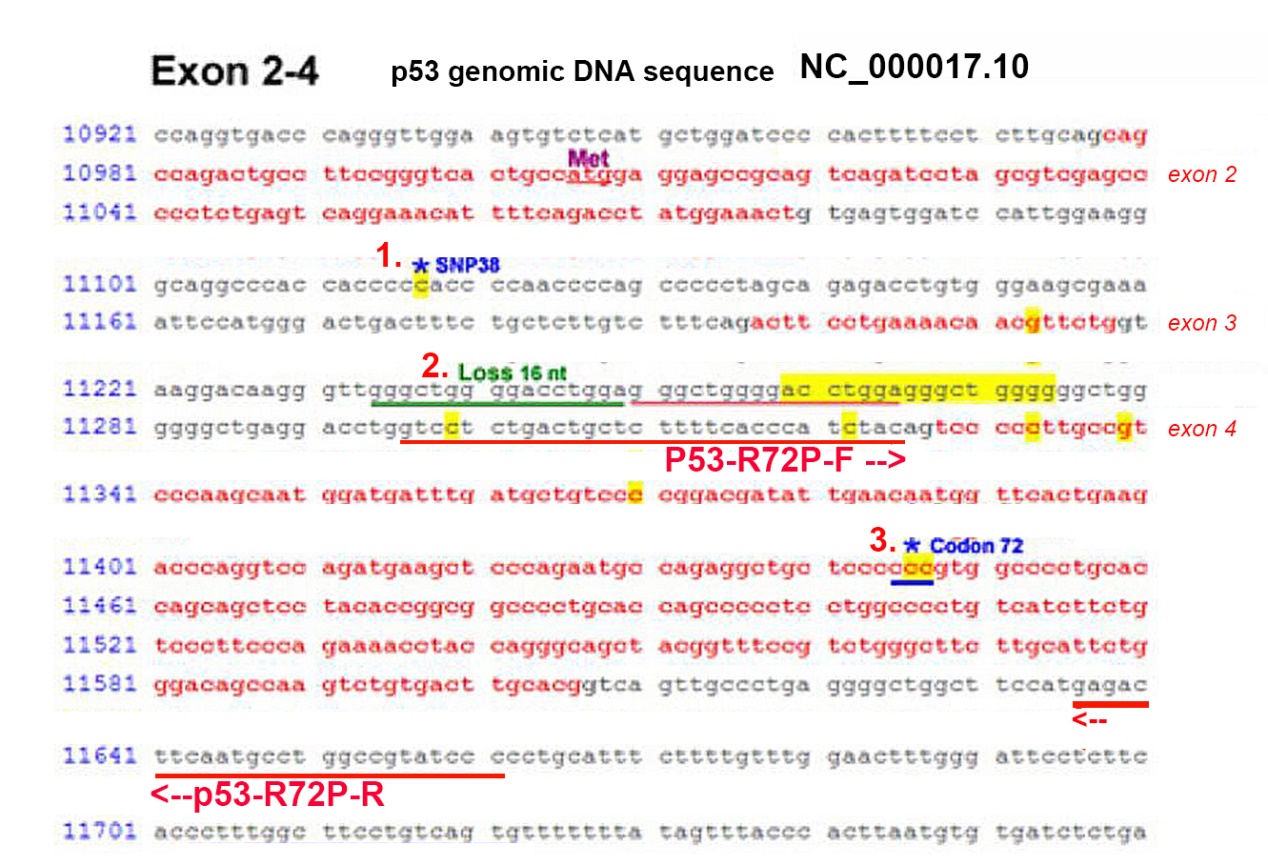


Fig. S2. The location sites of PCR-RFLP primers in p53 genomic DNA.


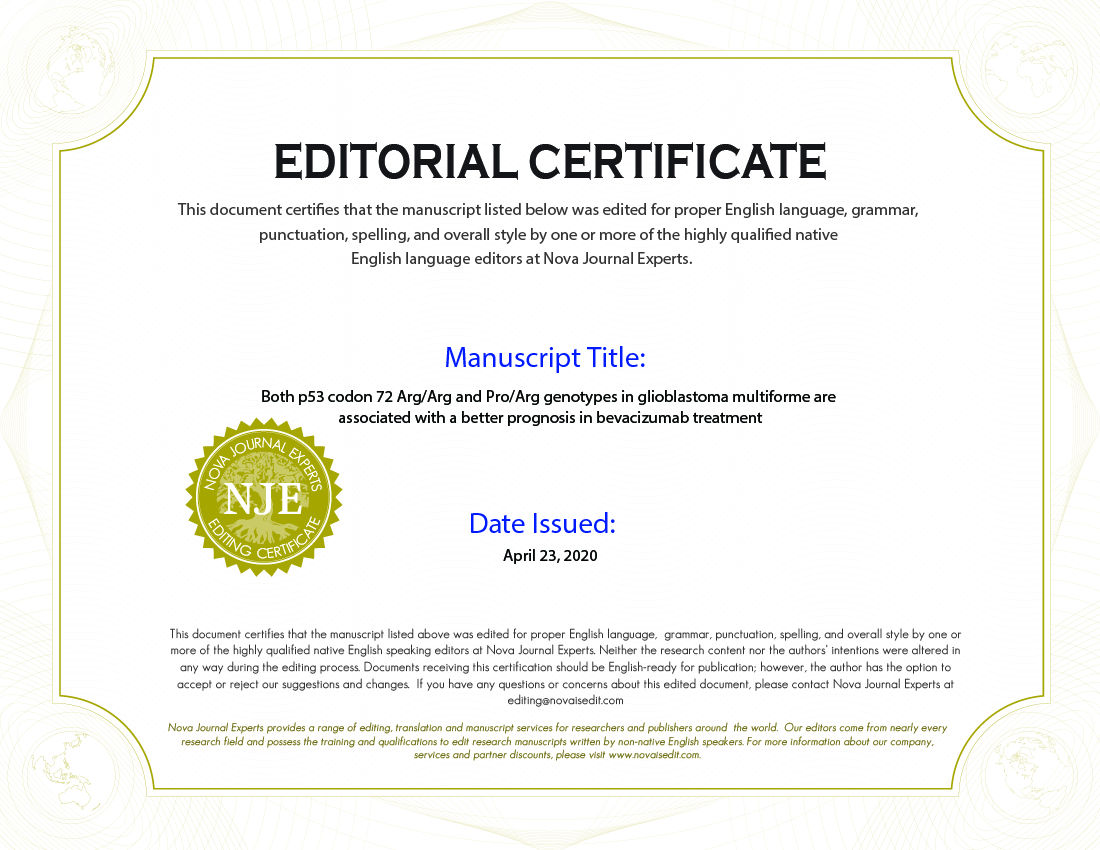


Fig. S3. The proof of this edited article by the team that specializes in English.
